# Control of aging by the renin–angiotensin system: a review of *C. elegans, Drosophila,* and mammals

**DOI:** 10.3389/fphar.2022.938650

**Published:** 2022-09-14

**Authors:** Brian M. Egan, Andrea Scharf, Franziska Pohl, Kerry Kornfeld

**Affiliations:** ^1^ Department of Developmental Biology, Washington University School of Medicine, St. Louis, MO, United States; ^2^ Department of Biological Sciences, Missouri University of Science and Technology, Rolla, MO, United States; ^3^ Department of Medicine, Washington University School of Medicine, St. Louis, MO, United States

**Keywords:** Captopril (ACE-I), longevity, acn-1, ACE-angiotensin-converting enzyme, Ance, Enalapril, Losartan, Lisinopril

## Abstract

The free-living, non-parasitic nematode *Caenorhabditis elegans* is a premier model organism for the study of aging and longevity due to its short lifespan, powerful genetic tools, and conservation of fundamental mechanisms with mammals. Approximately 70 percent of human genes have homologs in *C. elegans*, including many that encode proteins in pathways that influence aging. Numerous genetic pathways have been identified in *C. elegans* that affect lifespan, including the dietary restriction pathway, the insulin/insulin-like growth factor (IGF) signaling pathway, and the disruption of components of the mitochondrial electron transport chain. *C. elegans* is also a powerful system for performing drug screens, and many lifespan-extending compounds have been reported; notably, several FDA-approved medications extend the lifespan in *C. elegans*, raising the possibility that they can also extend the lifespan in humans. The renin–angiotensin system (RAS) in mammals is an endocrine system that regulates blood pressure and a paracrine system that acts in a wide range of tissues to control physiological processes; it is a popular target for drugs that reduce blood pressure, including angiotensin-converting enzyme (ACE) inhibitors and angiotensin II receptor blockers (ARBs). Emerging evidence indicates that this system influences aging. In *C. elegans*, decreasing the activity of the ACE homolog *acn-1* or treatment with the ACE-inhibitor Captopril significantly extends the lifespan. In *Drosophila*, treatment with ACE inhibitors extends the lifespan. In rodents, manipulating the RAS with genetic or pharmacological interventions can extend the lifespan. In humans, polymorphisms in the ACE gene are associated with extreme longevity. These results suggest the RAS plays a conserved role in controlling longevity. Here, we review studies of the RAS and aging, emphasizing the potential of *C. elegans* as a model for understanding the mechanism of lifespan control.

## Introduction

### *Caenorhabditis elegans* is a powerful model system for aging pharmacology

Age-related degenerative changes are a major issue for human health (Vijg and Campisi, 2008). A wide variety of systems are affected including the reproductive, central and peripheral nervous, musculoskeletal, immune, cardiac, renal, and respiratory. The age-related reduction of muscular strength, or sarcopenia, is a serious problem for many elderly people. Although the quest for methods to delay aging is a longstanding human endeavor, no pharmacological agents have yet been demonstrated to delay human aging. One of the biggest challenges to understanding and treating human aging is the fact that humans live so long and age so slowly. Thus, a critical need in aging research is model organisms that are short-lived, amenable to experimentation, and relevant to humans.

The analysis of genetically tractable model organisms with short lifespans, such as yeast, worms, and flies, has resulted in the identification of genes that can modulate longevity (Guarente and Kenyon, 2000). Studies of mice have provided substantial evidence that at least some mechanisms that affect the rate of age-related degeneration have been conserved during animal evolution (reviewed in Blagosklonny (2008), Vijg and Campisi (2008)). Thus, short-lived animals may provide meaningful guides to the biology of human aging and serve as the proving grounds where interventions that delay age-related degeneration can be identified and characterized. The report by the intervention testing program (ITP) of the National Institutes of Aging that Rapamycin extends the lifespan of mice is a compelling example, since Rapamycin was first demonstrated to influence the lifespan in yeast (Kaeberlein et al., 2005; Powers et al., 2006; Harrison et al., 2009).

*C. elegans* is a powerful and relevant experimental system that is excellent for the identification of genes and drugs that modulate the rate of aging. *C. elegans* is a free-living, hermaphroditic nematode that displays an extensive conservation of fundamental biological processes with other animals. Pioneering studies by Sydney Brenner and others established *C. elegans* as a powerful experimental model system for forward genetics, reverse genetics, and molecular analysis supported by a fully sequenced genome (*C. elegans* Sequencing Consortium, 1998; Brenner, 1974). *C. elegans* has been used to characterize fundamental and highly conserved biological processes such as RNA interference (RNAi) and apoptosis (Sulston and Horvitz, 1977; Fire et al., 1998). It has been used to develop innovative experimental techniques such as the *in vivo* expression of green fluorescent protein (GFP) (Chalfie et al., 1994); these and other discoveries have had a major impact on understanding mammalian biology. *C. elegans* is extremely well understood at the cellular anatomical level, since the entire cell lineage of the 959 somatic nuclei has been determined (Sulston and Horvitz, 1977).

*C. elegans* is excellent for studies of aging because the adults display the progressive, degenerative changes that are typical of aging in larger animals, but the adult lifespan is only about 15 days (Johnson, 1987). Age-related degenerative changes in *C. elegans* have been characterized extensively, and the genetic analysis of *C. elegans* has resulted in the discovery of genes and pathways that modulate longevity (Guarente and Kenyon, 2000; Kenyon, 2001). Many well-characterized mutations that influence aging by affecting known pathways are reagents that can be used to investigate the mechanism of action of newly discovered genes and drugs that influence aging. *C. elegans* has been used successfully to analyze the molecular targets of drugs (Carroll et al., 2003; Jones et al., 2005). Since nearly 75% of all *C. elegans* genes have human counterparts, there is a good chance that the molecular target of a drug in the worm will be similar to that in humans (*C. elegans* Sequencing Consortium, 1998).

The first of what is now a large class of ACE inhibitors, Captopril is an oligopeptide derivative developed in 1975 based on a peptide found in the venom of a snake, *Bothrops jararaca*, the Brazilian pit viper (Ondetti et al., 1977). ACE inhibitors modulate the renin–angiotensin system (RAS), a mechanism by which the body adapts to hypotension (Basso and Terragno, 2001; Igic and Igic, 2009). In this review, we focus on the emerging evidence from *C. elegans*, *Drosophila,* and mammals that the RAS controls longevity and drugs that target this system might be useful agents in the quest to extend human lifespan.

### The RAS is an endocrine system that controls blood pressure and a paracrine system that mediates a wide range of physiological processes

High blood pressure, or hypertension, is defined as a systolic blood pressure over 140 mmHg and/or diastolic blood pressure over 90 mmHg (Mills et al., 2020). It is a common malady throughout the world, affecting more than 31% of the adult population (∼1.4 billion people); the percentage of the population suffering from hypertension continues to increase, creating an accelerating health crisis (Mills et al., 2020). High blood pressure is associated with and a likely cause of many cardiovascular and renal diseases (Mills et al., 2020) and is estimated to result in more than 10 million deaths per year worldwide (Forouzanfar et al., 2017). What causes hypertension? Dietary factors such as sodium are one cause; genetics can be a second cause (Singh et al., 2012). Age has been identified as a major risk factor for hypertension, with incidences rising in aging populations (Singh et al., 2012). Understanding the interaction between age and hypertension is important for maintaining the health of an aging population.

**FIGURE 1:**
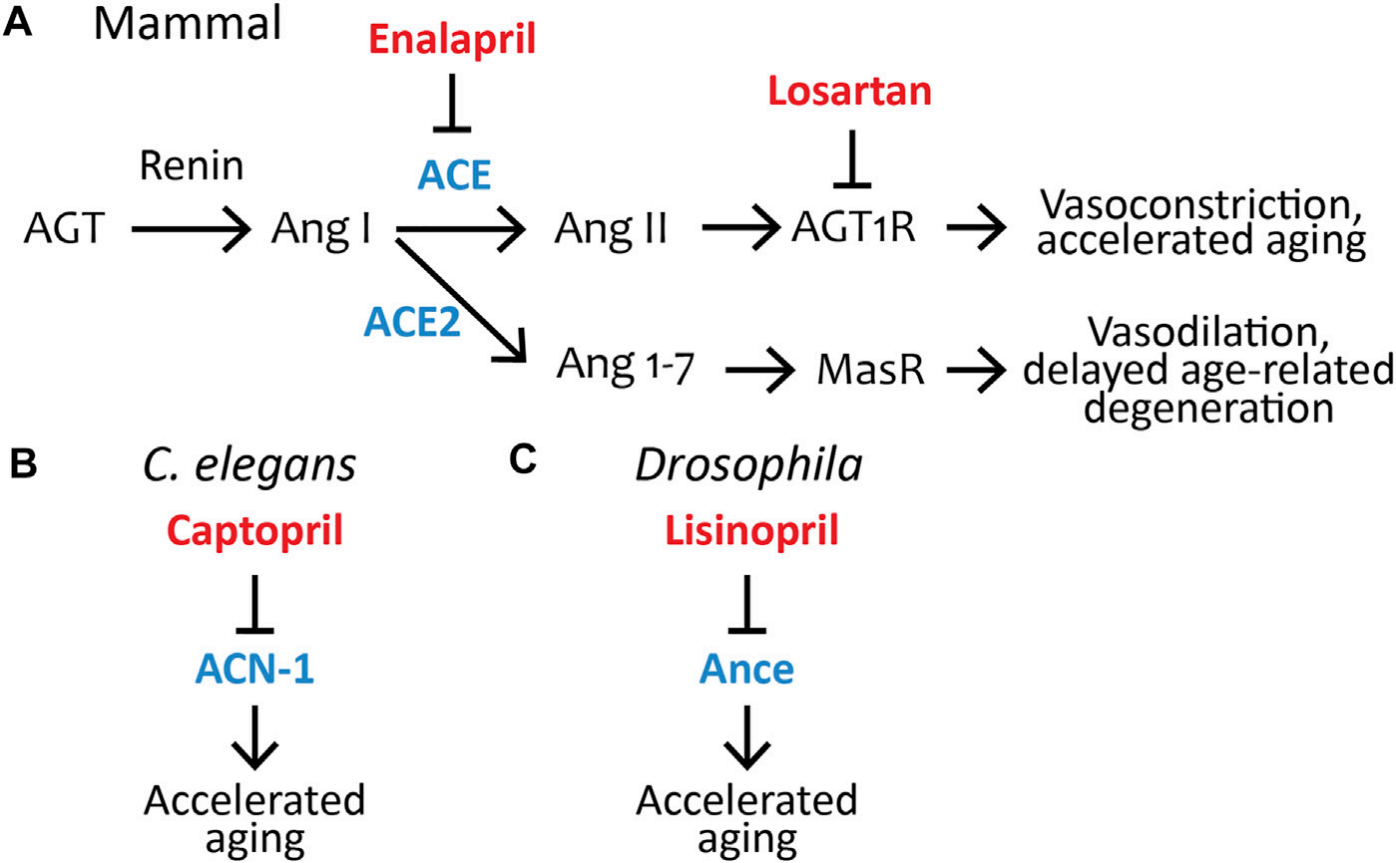
Pharmacological inhibition of the renin–angiotensin system influences aging in *C. elegans*, *Drosophila*, and mammals. **(A)** The RAS pathway in mammals; the ACE inhibitor Enalapril and angiotensin II type-1 receptor blocker Losartan are FDA-approved drugs that control aging in non-human mammals (see Table 1). AGT, Angiotensinogen; Ang I, Angiotensin I; ACE, Angiotensin-converting enzyme; Ang II, Angiotensin II; AGT1R, Angiotensin II type-1 receptor; ACE2, Angiotensin-converting enzyme 2; Ang 1-7, Angiotensin (1-7); MasR, Mas Receptor. **(B)** The FDA-approved ACE-inhibitor drug Captopril inhibits *acn-1,* the only *C. elegans* ACE homolog, to control aging (Kumar et al., 2016). ACN-1, Angiotensin-converting enzyme-like non-peptidase. **(C)** The FDA-approved ACE inhibitor drug Lisinopril inhibits Ance, the *Drosophila* ACE homolog, to control aging (Gabrawy et al., 2019). Ance, ANgiotensin-converting enzyme. Blue, ACE and its homologs ACE2, Ance, and ACN-1; red, ACE inhibitors (Captopril, Enalapril, Lisinopril) or ARBs (Losartan), which have been shown to influence aging.

The RAS was first identified as a crucial regulator of blood pressure in the mid-20th century ((Skeggs et al., 1956), reviewed in Basso and Terragno (2001), Igic and Igic (2009)). The classic understanding was that the RAS is an endocrine system. In response to low blood pressure, the kidney secretes renin, an aspartyl protease. Renin cleaves the peptide angiotensinogen (AGT), which the liver releases into the blood stream, into angiotensin I (Ang I), a 10 amino acid peptide. Ang I is cleaved into angiotensin II (Ang II), an eight-amino acid peptide, by the angiotensin-converting enzyme (ACE), a metalloprotease secreted by the lungs. Ang II binds to the angiotensin II type 1 receptor (AGT1R) on vascular endothelial cells. AGT1R is a G-protein-coupled receptor that initiates a signal transduction pathway resulting in vasoconstriction and increased blood pressure (Figure 1A) (reviewed in Peach (1977), Reid et al. (1978), Fyhrquist and Saijonmaa (2008), and Santos et al. (2019)). In the last 30 years, exciting new discoveries have shown that the RAS is significantly more complicated in two dimensions: the endocrine system has many additional components, including some that promote vasodilation, and the RAS functions as a paracrine/autocrine system in many organs to mediate a wide range of physiological responses.

In 2000, a second isoform of ACE was described in mammals, called ACE2 (Lavoie and Sigmund, 2003). ACE2 is a transmembrane protein that cleaves Ang I or Ang II into the heptapeptide angiotensin (1-7) (Ang(1-7)). Furthermore, the receptor for Ang(1-7) was discovered, called the Mas receptor (MasR). Activation of the Mas receptor results in vasodilation (Figure 1A) (Santos et al., 2003). The recent discovery that ACE2 appears to be the main entry point into cells for the SARS-CoV-2 coronavirus, the cause of the COVID-19 pandemic, has resulted in intense scrutiny (Wrapp et al., 2019). Additionally, ACE2 has been proposed to influence aging since inhibition accelerates age-related degenerative changes (Takeshita et al., 2018; Nozato et al., 2019; Takeshita et al., 2020). Other discoveries include the (pro)renin receptor [(P)RR], which binds and activates renin in tissues, and the angiotensin II type 2 receptor (AGT2R) on vascular endothelial cells. AGT2R is a G-protein-coupled receptor that initiates a signal transduction pathway resulting in vasodilation and reduced blood pressure. Thus, Ang II can increase blood pressure by acting through the AGT1R receptor and reduce blood pressure by acting through the AGT2R receptor, and the expression levels of the two receptors play an important role in the overall response (Figure 1A).

The receptors for angiotensin peptides are expressed in a wide range of tissues, indicating that they function in paracrine/autocrine systems controlled by the local generation of peptide agonists (reviewed in Lavoie and Sigmund, 2003; Bader, 2010; Nehme et al., 2019). The brain expresses the AGT1R and AGT2R receptors, which influence the autonomic nervous system, the hypothalamus–pituitary axis, vasopressin release, baroreflex sensitivity, and thirst and salt appetite. The overall effects of brain activation are to increase blood volume and blood pressure, but are also implicated in higher brain functions such as anxiety and stress (Abiodun and Ola, 2020; Balthazar et al., 2021). In the kidney, local RAS signaling plays a role in kidney development and renal function in adults. In the heart, Ang II acts through the AGT1R to induce cardiac hypertrophy and fibrosis and through the AGT2R to cause the opposite effects. In the gastrointestinal system, the RAS regulates intestinal physiological functions including electrolyte homeostasis, digestion, peptide transport, glucose, sodium and water absorption, and gastrointestinal motility (Jaworska et al., 2021). The RAS is important during pregnancy and acts locally in the uteroplacental unit to mediate angiogenesis, trophoblastic invasion, and adequate placentation (Leal et al., 2022). It is possible that the tissue-specific functions of the RAS represent primordial functions and are the target of interventions that modulate aging.

**FIGURE 2:**
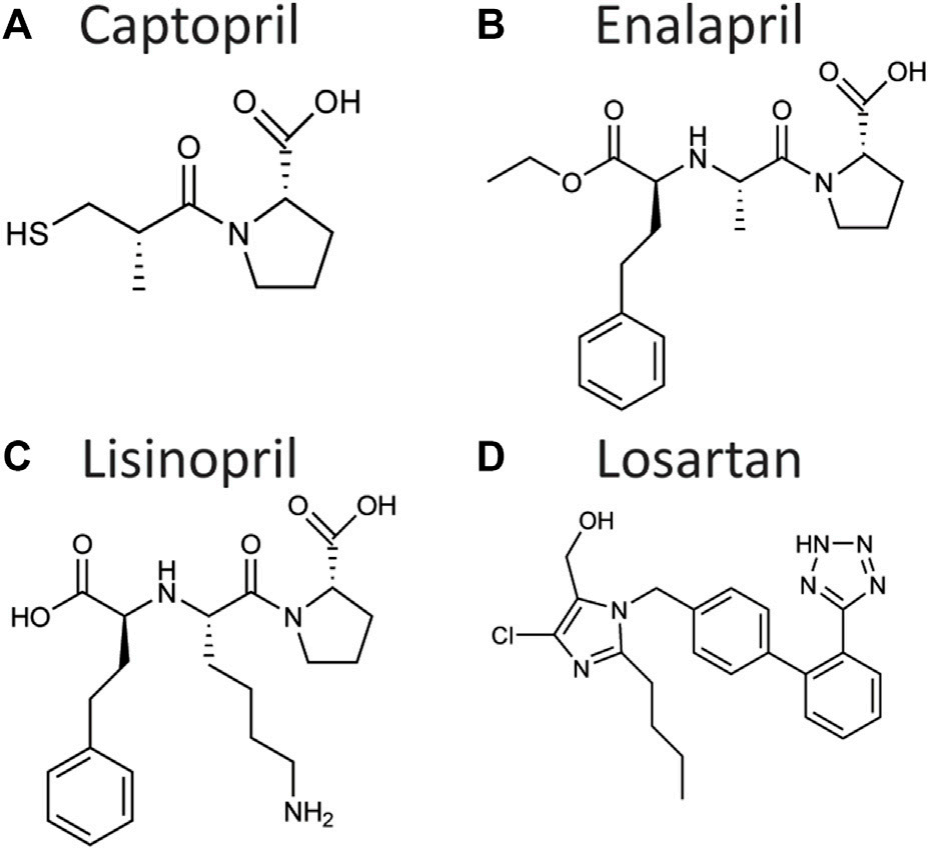
Chemical structure of the angiotensin-converting enzyme inhibitors Captopril **(A)**, Enalapril **(B)**, and Lisinopril **(C)**, and of the angiotensin II receptor blocker Losartan **(D)**. These drugs have been shown to control aging in *C. elegans*, *Drosophila*, and/or rodents.

A major breakthrough in the treatment of hypertension was the development of Captopril, the first ACE inhibitor compound, in 1975 (Figure 2A) (Cushman and Ondetti, 1991). Observations of the hypotensive effects of venom of the snake *Bothrops jararaca* made it clear that ACE was a critical regulator of blood pressure and a useful target for inhibition (Cushman and Ondetti, 1991). Rational drug design techniques allowed the creation of Captopril as a peptide-analogue targeting and inhibiting the active site of ACE, with later generations of ACE inhibitors, such as Enalapril (Figure 2B) or Lisinopril (Figure 2C), improving the activity and bioavailability. Further research into the RAS led to the development of angiotensin II receptor blockers (ARBs) such as Losartan (Figure 2D) (Duncia et al., 1990). More recently, renin inhibitors such as Aliskiren were developed (Jensen et al., 2008).

The blood pressure regulation activity of the RAS is well characterized and has been studied in many mammalian model organisms (reviewed in (Peach, 1977; Reid et al., 1978; Igic and Igic, 2009; Santos et al., 2019). However, components of the RAS have also been discovered in non-mammalian organisms that lack closed circulatory systems. ACE homologs have been identified in organisms throughout the Bilaterian clade, including the *C. elegans* homolog *acn-1* and the *Drosophila* homolog *Ance* (Figures 1B,C). Functional ACE homologs have also been identified in insects, crustaceans, annelids, and mollusks (reviewed in (Fournier et al., 2021)). Based on its presence in such diverse phyla, it is likely that ACE first evolved in a common ancestor roughly 600 million years ago (Dos Reis et al., 2015), prior to the evolution of the animal closed circulatory system (Simões-Costa et al., 2005). These observations raise an intriguing question: What is the ancestral function of ACE? Insight into that function can help address the role of the RAS during aging.

**FIGURE 3:**
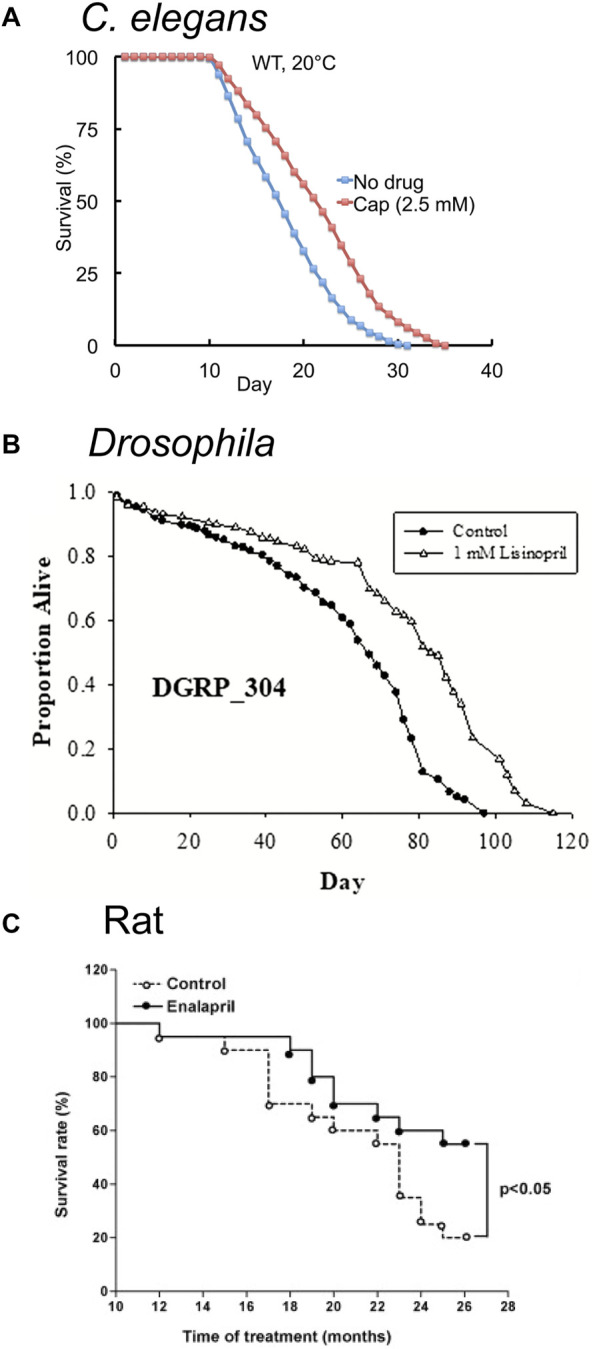
ACE inhibitors extend lifespan in model organisms. **(A)** The FDA-approved ACE inhibitor drug Captopril (cap) extends adult lifespan in *C. elegans* by about 21%. Survival curves of wild-type (WT) hermaphrodites treated with 2.5 mM Captopril from the L4 stage onward at 20°C. Adapted from (Kumar et al., 2016). **(B)** The FDA-approved ACE inhibitor drug Lisinopril extends lifespan in *Drosophila melanogaster*. Survival curve of populations of the *Drosophila* Genetic Reference Panel (DGRP, strain DGRP_304 is depicted) exposed to 1 mM Lisinopril. Adapted from (Gabrawy et al., 2019). **(C)** The FDA-approved ACE-inhibitor drug Enalapril extends lifespan in adult rats. Survival curves of adult Wistar rats exposed to 10 mg/kg Enalapril in water for 26 months. Enalapril treatment reduced mortality by 45% in the 26 month period. Adapted from (Santos et al., 2009).

**FIGURE 4:**
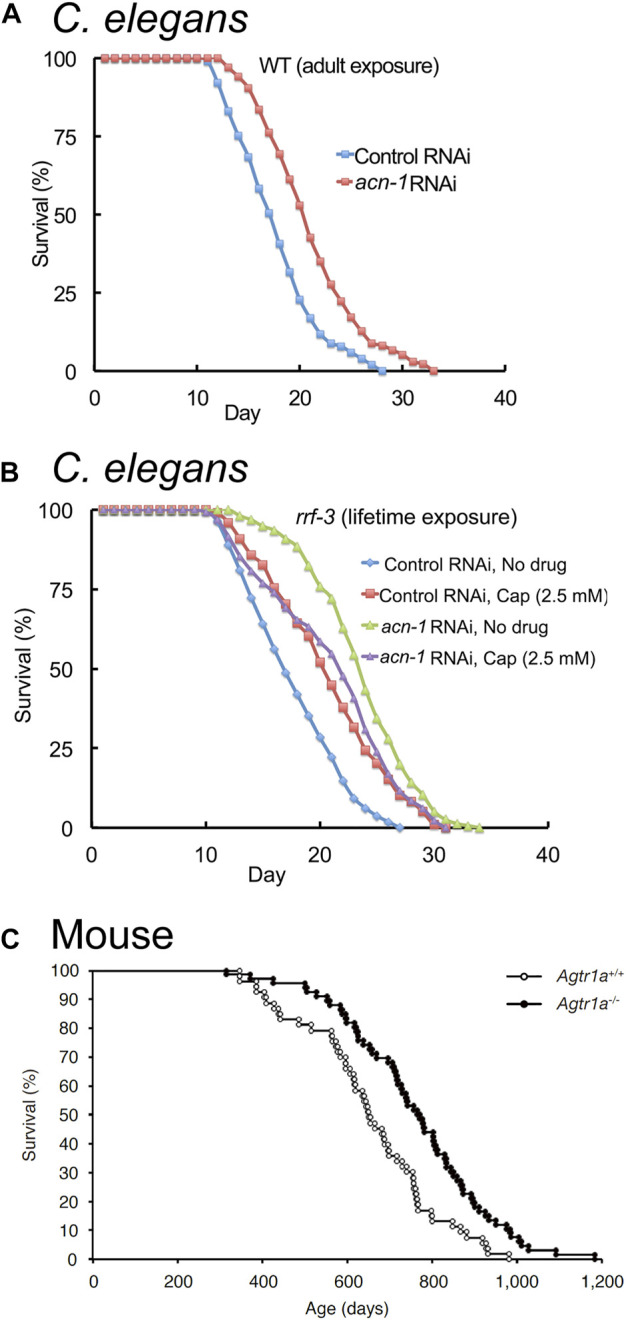
Reducing ACE expression extends lifespan in *C. elegans* and mice. **(A)** Reducing *acn-1* expression via RNA-interference (RNAi) extends adult lifespan in *C. elegans* by about 20%. Survival curves of wild-type (WT) hermaphrodites exposed to *acn-1* or control dsRNA-expressing bacteria from the L4 stage onward at 20°C. **(B)** Captopril and *acn-1* RNAi are not additive in extending the lifespan of adult *C. elegans*, suggesting that Captopril inhibits *acn-1* to control aging. Survival curves of RNAi-hypersensitive strain *rrf-3(pk1426)* exposed to *E. coli* bacteria expressing control or *acn-1* dsRNA from the embryonic stage and to 2.5 mM Captopril from L4 stage at 20°C. **(A,B)** Adapted from (Kumar et al., 2016). **(C)** Knock-out of AGT1R extends adult lifespan in mice by about 17%. Survival curves of Agtr1a^−/−^ and Agtr1a^+/+^ mice (Sugaya et al., 1995) fed with a standard diet. Adapted from (Yabumoto et al., 2015).

Emerging evidence has identified important connections between the RAS and aging. Pharmacological or genetic inhibition of members of the RAS can extend lifespan and delay age-related degenerative changes in mice and rats (Figures 3C, 4C) (reviewed in (Basso et al., 2005; de Cavanagh et al., 2011; Capettini et al., 2012; Kamo et al., 2016; Mogi, 2020; Jaworska et al., 2021; Le et al., 2021)). The study of ACE and the RAS in the context of aging is complicated in mammalian systems due to their pleiotropic effects not only on aging but also on vasoconstriction, renal, cardiovascular, and pulmonary health and function, and less understood functions in the brain (reviewed in (Le et al., 2021)). In contrast, non-mammalian organisms lacking closed circulatory systems can be used to distinguish the aging effects of the RAS from the cardiovascular functions.

### Captopril and the *acn-1* gene influence aging in *Caenorhabditis elegans*

The nematode ACE homolog *acn-1* was first identified by Brooks et al. (2003) as necessary for larval development and adult morphogenesis. Green fluorescent protein (GFP)-tagged ACN-1 driven by an *acn-1* promoter was expressed in the embryo hypodermis, hypodermal seam cells, and vulva of the L4 hermaphrodite, and the ray papillae in the L4 male tail. *acn-1* RNAi-mediated knockdown resulted in larval molting defects, characterized by a failure to shed the cuticle. Gonad injection of dsRNA resulted in larval arrest at L2 and an early mortality phenotype, indicating that it causes a strong reduction of function and *acn-1* is necessary for larval survival; feeding of dsRNA-expressing bacteria allows many animals to survive the larval stage, indicating a less severe reduction of function, and animals display cuticle shedding defects in the L3/L4 and L4/adult molts. RNAi-mediated knockdown of *nhr-23* and *nhr-25* reduced the expression of ACN-1, suggesting that these genes regulate *acn-1.* Loss of function of these two genes was previously reported to induce cuticle shedding defects similar to *acn-1* loss of function (Gissendanner and Sluder, 2000). Indeed, a comprehensive RNAi screen by Frand et al. (2005) identified *acn-1*, along with *nhr-23* and *nhr-25*, as inducing molting defects. Based on these results, *acn-1* appears to act in larval animals to regulate molting downstream of *nhr-23* and *nhr-25*.

Oskouian et al. (2005) replicated the finding of Brooks et al. (2003) that the injection of worms with *acn-1* dsRNA resulted in the arrest of larval development. Kumar et al. (2016) did not observe significant larval arrest after *acn-1* RNAi feeding treatment. The difference may be due to the method of administration (feeding in the case of Kumar et al. (2016), and injection into the gonad in the cases of Oskouian et al. (2005), Brooks et al. (2003).

The heterochronic pathway controls the timing of molting and other development events by time-dependent expression of genes including several microRNAs. Knockdown of heterochronic pathway components disrupts crucial timing in cell fate decisions, leading to precocious or retarded cell fate phenotypes. Knockdown of *acn-1* suppresses the ectopic phenotypes associated with the knockdown of the heterochronic pathway microRNA *let-7*, and *acn-1* expression is modulated throughout the L1 stage in a manner dependent on *lin-41* and *hbl-1,* both targets of *let-7* (Metheetrairut et al., 2017). Thus, *acn-1* genetically interacts with several components of the heterochronic pathway. Ahn et al. (2006) found that *acn-1* transcription was downregulated in *tax-6(jh107)* mutants. *tax-6* is the nematode homolog of the mammalian calcineurin A subunit, and the *jh107* allele represents a constitutively active gain-of-function mutation. This suggests a role for *tax-6* in the regulation of *acn-1* protein expression. Dubois et al. (2019) found that ACN-1 protein expression was affected by exposure to ionizing gamma radiation; protein expression was upregulated following chronic exposure and down-regulated following acute exposure.

Based on sequence alignments, the predicted ACN-1 protein lacks several histidine residues that are ligands for the zinc cofactor necessary for the metalloprotease activity of ACE, suggesting that ACN-1 protein lacks this enzyme activity (Brooks et al., 2003). Biochemical analyses of the catalytic activity of ACN-1 have not been performed, so it remains unknown if ACN-1 has the metalloprotease activity observed in ACE. It has been speculated that ACN-1 may function by binding to and sequestering its substrate, but further research is needed to determine the biochemical function of ACN-1.

While *acn-1* function in larval animals is well established, Kumar et al. (2016) showed that *acn-1* RNAi treatment initiated after the L4 stage, when the animal has completed all of its larval molts, extends lifespan and delays age-related degenerative changes (Figure 4A; Table 2). This suggests that *acn-1* functions in adults to control aging. Further research is required to better understand the function of *acn-1* in adult animals.

Kumar et al. (2016) performed a screen for FDA-approved medications that extend lifespan in *C. elegans*. Captopril, an ACE inhibitor, extended lifespan in hermaphrodite worms by more than 30% (Figure 3A; Table 1); additionally, Captopril treatment delayed age-related degeneration in multiple systems, including body movement and pharyngeal pumping rate. Captopril treatment increased thermotolerance and oxidative stress resistance, phenotypes associated with extended longevity (Kumar et al., 2016). The lifespan extension phenotype appeared to be independent of several known longevity pathways, including the caloric restriction, mitochondrial dysfunction, sirtuin, and TOR signaling pathways.

**TABLE 1:** Pharmacological inhibition of the RAS system and effects on age-related phenotypes.

Drug name^a^	Animal^b^	Age-related phenotype(s)^c^	Dose/notes^d^
**Captopril** (ACE inhibitor)	Worm	Lifespan ↑ (Kumar et al., 2016; Kucuktepe, 2021), pharyngeal pumping rate ↑ (Kumar et al., 2016; Kucuktepe, 2021), body movement rate ↑ (Kumar et al., 2016; Kucuktepe, 2021), thermotolerance ↑ (Kumar et al., 2016; Kucuktepe, 2021), oxidative stress resistance ↑ (Kumar et al., 2016; Kucuktepe, 2021), lipid storage ↓ (Kucuktepe, 2021)	2.5 mM in agar; hermaphrodites only
**Lisinopril** (ACE inhibitor)	Fly	Lifespan ↑ (Gabrawy et al., 2019), protein aggregation ↓ (Gabrawy et al., 2019), climbing speed ↑ (Crocco et al., 2022), endurance ↑ (Crocco et al., 2022), strength ↑ (Crocco et al., 2022), mitochondrial number ↑ (Ederer et al., 2018), starvation resistance ↓ (Ederer et al., 2018)	1 mM in feed; males only; effects are genotype-specific
**Enalapril** (ACE inhibitor)	Mouse	(1) Body weight ↑ (Ferder et al., 1994; Inserra et al., 1995), mitochondrial number ↑ (Ferder et al., 1994), kidney mass ↑ (Ferder et al., 1994), glomerular number and size ↑ (Ferder et al., 1994), glomerulosclerosis ↓ (Ferder et al., 1994), serum potassium ↑ (Inserra et al., 1995), myocardiosclerosis ↓ (Inserra et al., 1995)	(1) 5, 10, or 20 mg/L in water; females only
		(2) Frailty ↓, systolic blood pressure ↓ (Keller et al., 2019)	(2) 30 mg/kg/day in feed; males and females
**Enalapril** (ACE inhibitor)	Rat	(1) Lifespan ↑ (Santos et al., 2009), body weight ↓(Santos et al., 2009; González Bosc et al., 2000; Basso et al., 2007), caloric intake ↓ (Santos et al., 2009), systolic blood pressure ↓ (González Bosc et al., 2000; de Cavanagh et al., 2003; Basso et al., 2007; de Cavanagh et al., 2008; Santos et al., 2009), water intake ↑ (González Bosc et al., 2000; Basso et al., 2007), heart weight ↓ (González Bosc et al., 2000; Basso et al., 2007), cardiac health ↑ (González Bosc et al., 2000; Basso et al., 2007), renal health and function ↑ (de Cavanagh et al., 2003; de Cavanagh et al., 2008), kidney degradation ↓ (de Cavanagh et al., 2003; de Cavanagh et al., 2008), glomerular size ↓ (de Cavanagh et al., 2003; de Cavanagh et al., 2008), proteinuria ↓ (de Cavanagh et al., 2003; Carter et al., 2004; Inserra et al., 2009), mitochondrial function ↑ (de Cavanagh et al., 2003; de Cavanagh et al., 2008), glomerular size ↓ (Inserra et al., 2009)	(1) 10 mg/kg/day in water; males only
		(2) Heart rate ↑ (Carter et al., 2004), blood pressure ↓ (Carter et al., 2004), body fat ↓ (Carter et al., 2004), grip strength ↑ (Carter et al., 2004), climbing of incline ↑ (Carter et al., 2004)	(2) 40 or 80 mg/kg/day by subcutaneous injection; males and females
		(3) Body weight ↓ (Carter et al., 2011), fat mass ↓ (Carter et al., 2011), grip strength ↑ (Carter et al., 2011), food consumption ↓ (Carter et al., 2011), movement ↑ (Carter et al., 2011), tumor incidence ↓ (Carter et al., 2011)	(3) 40 mg/kg/day in feed; males only
**Losartan** (Angiotensin II type 1 receptor blocker)	Rat	(1) Systolic blood pressure ↓ (González Bosc et al., 2000), water intake ↓ (González Bosc et al., 2000), heart weight ↓ (González Bosc et al., 2000), cardiac health ↑ (González Bosc et al., 2000)	(1) 10 mg/kg/day in water; males only
		(2) Systolic blood pressure ↓ (de Cavanagh et al., 2003; Basso et al., 2007; de Cavanagh et al., 2008), renal health and function ↑ (de Cavanagh et al., 2003; de Cavanagh et al., 2008), kidney degradation ↓ (de Cavanagh et al., 2003; de Cavanagh et al., 2008; Inserra et al., 2009), glomerular size ↓ (de Cavanagh et al., 2003; de Cavanagh et al., 2008; Inserra et al., 2009), proteinuria ↓(de Cavanagh et al., 2003; de Cavanagh et al., 2008; Inserra et al., 2009), heart weight ↓ (Basso et al., 2007), cardiac health ↑ (de Cavanagh et al., 2003; de Cavanagh et al., 2008), mitochondrial number and function ↑ (de Cavanagh et al., 2003; de Cavanagh et al., 2008)	(2) 30 mg/kg/day in water; males only
		(3) Grip strength ↑ (Carter et al., 2011)	(3) 30 mg/kg/day in feed; males only

a **Compound name** (protein target).

b Worm, *Caenorhabditis elegans;* fly, *Drosophila melanogaster*; mouse, *Mus musculus*; rat, *Rattus norvegicus*.

c Phenotypes display age-related change; arrow indicates the direction of drug effect with respect to the untreated age-matched cohort.

d Drug dose; route of administration; sex of animals; notes.

Kucuktepe (2021) treated animals with Captopril and replicated the lifespan extension, delayed the decline of pharyngeal pumping rate, increased thermotolerance, and increased oxidative stress resistance phenotypes reported by Kumar et al. (2016). Kucuktepe (2021) examined the effect of Captopril administration on lipid levels; triglyceride levels were significantly reduced based on oil red O-staining of L4-stage worms. The primary effect was on intestinal lipid droplets, which were reduced in diameter. Biochemical analysis indicated a reduction in triglycerides and protein content after Captopril treatment.

Kumar et al. (2016), Kucuktepe (2021) both demonstrated that the lifespan extension caused by Captopril treatment was abrogated by a *daf-16* loss-of-function mutation. The DAF-16 transcription factor is the terminus of the insulin/insulin-like growth factor (IGF) signaling pathway, a well-characterized pathway that influences aging (Kimura et al., 1997; Tissenbaum and Ruvkun, 1998). Kumar et al. (2016) reported that DAF-16 does not display nuclear localization upon treatment with Captopril. Furthermore, Kucuktepe (2021) observed that the body fat reduction caused by Captopril in wild-type animals was not displayed by *daf-16(lf)* mutants, suggesting that both lifespan extension and body fat reduction are DAF-16-dependent. Further research is needed to determine the nature of the Captopril–DAF-16 interaction.

Kumar et al. (2016) examined interactions of the ACE inhibitor Captopril with the *acn-1* gene. RNAi-mediated inhibition of *acn-1* caused many phenotypes similar to Captopril administration, including lifespan extension (Figure 4A). Furthermore, RNAi-mediated knockdown of *acn-1* did not further enhance the lifespan extension caused by Captopril treatment (Figure 4B), suggesting that Captopril affects aging by the inhibition of *acn-1*. In contrast to Kumar et al. (2016), Dalton and Curran (2018) reported that treatment with acn-1 RNAi decreased heat and oxidative stress resistance (Kumar et al., 2016; Dalton and Curran, 2018). These results may be explained by differences in methodology, as the two studies differed in the timing of temperature shift (larval day 1 or 2 in Dalton and Curran (2018) and adult day 3 in Kumar et al., (2016)) and the timing of *acn-1* RNAi administration (beginning at hatch for Dalton and Curran (2018) and beginning at L4 for Kumar et al. (2016)).

The Greenwald group generated a compendium of *C. elegans* genes with homologs in humans, called “Ortholist” (Shaye and Greenwald, 2011; Kim et al., 2018). Based on protein homology, nematode ACN-1 has homologs throughout the Bilaterian clade: in addition to humans, non-human primates, and rodents, ACE homologs are present in Zebrafish, *Xenopus*, *Drosophila*, and various other arthropods, suggesting that ACE-like proteins have been retained throughout many animal phyla for at least six-hundred million years of evolutionary history. Moreover, this suggests that the evolution of the ACE protein predated the evolution of the animal circulatory system, implying that the ancestral function of the ACE protein was unrelated to the regulation of blood pressure, especially considering its continued retention in phyla which lack closed circulatory systems. This is further reinforced by the apparent lack of functional homologs to the primary substrate of mammalian ACE, Angiotensinogen, in worms or *Drosophila*, as well as the lack of functional analogs to mammalian renin or the angiotensinogen receptors in these model organisms. Thus, it is apparent that the ancestral function of ACE was unrelated to blood pressure regulation and that this activity evolved later.

### Lisinopril and Ance affect aging in *Drosophila melanogaster*

The fruit fly *Drosophila melanogaster* is a valuable model organism for studies of genetics, development, behavior, and aging, having been commonly used for over a century (Castle, 1906; Morgan, 1910; Dunn, 1964; Roberts, 2006). Low maintenance costs, rapid generation time, well-developed genetic techniques, and relatively short lifespans make *Drosophila* one of the predominant model organisms for aging studies. Several studies have identified pathways that regulate aging including dietary restriction (DR), IGF signaling pathway, and the disruption of components of the mitochondrial electron transport chain (Broughton et al., 2005; Copeland et al., 2009; Altintas et al., 2016). *Drosophila* has been used to test anti-aging pharmacological interventions due to the availability of high-throughput screening techniques (Giacomotto and Ségalat, 2010; Jafari, 2010; Pandey and Nichols, 2011; Willoughby et al., 2013; Lee and Min, 2019). Compared to *C. elegans, Drosophila* has greater anatomical similarity to mammals (Copeland et al., 2009; Pandey and Nichols, 2011).

The *Drosophila* genome encodes two primary homologs of ACE as well as several that are more diverged. *Ance* (ANgiotensin-Converting Enzyme) is most similar to mammalian ACE (Figure 1C); *Ance* is expressed throughout the lifespan and plays roles in development and fertility (Houard et al., 1998; Hurst et al., 2003). *Acer* (angiotensin-converting enzyme-related) is most similar to mammalian ACE2 and plays roles in heart morphogenesis (Crackower et al., 2002). Several other homologous genes, *Ance-2* through *Ance-5*, likely resulted from gene duplication events; these genes do not have established functions and are not predicted to encode catalytically active proteins based on amino acid sequence (Coates et al., 2000). Ance and Acer, in contrast to ACN-1*,* have highly conserved residues in the active site residues similar to mammalian ACE. These proteins have been demonstrated to be catalytically active, since they can cleave Ang I in purified extracts (Cornell et al., 1995; Coates et al., 2000). ACE inhibitors bind to and inhibit the enzymatic activity of *Acer* and *Ance* (Cornell et al., 1995; Akif et al., 2010; Lee et al., 2020).

In agreement with results in worms, RNAi-mediated knockdown of *Ance*, the primary ACE homolog in *Drosophila*, extended lifespan (Gabrawy et al., 2019) (Table 2). Lisinopril treatment failed to enhance this effect, suggesting that Lisinopril extends lifespan via the inhibition of *Ance* (Gabrawy et al., 2019). In contrast, RNAi-mediated knockdown of the ACE2 homolog *Acer* reduces lifespan. Liao et al. (2013) used tissue-specific RNAi-mediated knockdown to reduce *Acer* activity in mesoderm and cardiac tissue, resulting in a reduction in lifespan, an increase in heart failure rate, a reduction in heart rate and the fractional shortening of the cardiac tissue, and an increase in the systolic and diastolic diameters. In agreement with this finding, Glover et al. (2019) observed that the genetic knockout of *Acer* reduced lifespan when fed *ad libitum*, but that starvation abrogated this reduction (Table 2).

**TABLE 2:** Genetic interventions on the RAS system and effects on age-related phenotypes.

Gene^a^	Intervention^b^	Animal^c^	Age-related phenotype(s)^d^	Notes^e^
*acn-1*	RNAi knockdown	Worm	Lifespan ↑ (Kumar et al., 2016), pharyngeal pumping rate ↑ (Kumar et al., 2016), body movement rate ↑ (Kumar et al., 2016), thermotolerance ↑ (Kumar et al., 2016), oxidative stress resistance ↑ (Kumar et al., 2016)	Hermaphrodites only
*Ance*	RNAi knockdown	Fly	Lifespan ↑ (Gabrawy et al., 2019)	Males only; effect is genotype-specific
*Acer*	RNAi knockdown	Fly	Lifespan ↓ (Liao et al., 2013), heart rate ↓ (Liao et al., 2013), heart failure rate ↑ (Liao et al., 2013), end-systolic diameter ↑ (Liao et al., 2013), end diastolic diameter ↑ (Liao et al., 2013), fractional shortening ↓ (Liao et al., 2013)	Males only; tissue-specific RNAi knockdown in mesoderm and cardiac tissue
*Acer*	Chromosomal mutation (Null)	Fly	Lifespan ↓ (Carhan et al., 2011; Glover et al., 2019)	Females only
Agt1r	Chromosomal mutation (Null)	Mouse	Lifespan ↑ (Benigni et al., 2009; Yabumoto et al., 2015), cardiac health and function ↑ (Benigni et al., 2009), mitochondrial number and function ↑ (Benigni et al., 2009), hair growth ↑ (Yabumoto et al., 2015), skin thickness ↑ (Yabumoto et al., 2015), fat layer thickness ↑ (Yabumoto et al., 2015), grip strength ↑ (Yabumoto et al., 2015), muscle repair ↑ (Yabumoto et al., 2015)	Males only
ACE2	Chromosomal mutation (Null)	Mouse	Grip strength ↓ (Takeshita et al., 2018), running distance ↓ (Takeshita et al., 2018), body weight ↓ (Takeshita et al., 2018), maximal muscle force ↓ (Takeshita et al., 2018)	Males only

a *Ance* is homologous to ACE; *Acer* is homologous to ACE2; *acn-1* is the only known ACE homolog in worms; Agt1r, Angiotensin II, type-1 receptor; ACE2, Angiotensin-converting enzyme 2.

b RNA-interference (RNAi) transiently reduces gene activity; chromosomal mutations appear to be null alleles.

c Worm, *Caenorhabditis elegans;* fly, *Drosophila melanogaster*; mouse, *Mus musculus*.

d Phenotypes display age-related change; arrow indicates the direction of effect with respect to wild-type or untreated age-matched cohort for chromosomal mutation or RNAi-mediated knockdown, respectively.

e RNAi affects either whole animal or specific tissues; sex of animal.

Gabrawy et al. (2019) examined the effect of the ACE inhibitor Lisinopril (Figure 2C) on several strains of *Drosophila*; in addition to extending lifespan (Figure 3B), Lisinopril increased the physical performance in aged flies, as measured by climbing speed, strength, and endurance (Table 1). Lisinopril treatment reduced aberrant protein aggregation in the muscles of aged flies (Gabrawy et al., 2019). Lisinopril extended lifespan in three genetic backgrounds, although the degree of extension varied. However, the physical performance and protein aggregation in aged flies varied in the degree by which they were influenced by Lisinopril, suggesting that genotype can affect phenotypes caused by Lisinopril in a complex manner.

Ederer et al. (2018) reported a genotype-specific effect of Lisinopril on metabolism; Lisinopril lowered the mitochondrial oxygen consumption in young flies, increased the number of mitochondria in aged flies, and reduced peroxide levels in young flies—however, each of these phenotypes occurred in strains with different genotypes. Genotype-specific effects were also observed in thoracic metabolite concentrations upon treatment with Lisinopril.

Crocco et al. (2022) expanded on this work using genome-wide association studies (GWAS) on 126 *Drosophila* strains with different genotypes treated with Lisinopril; while the majority of strains displayed extended lifespan and reduced age-related physical decline, some strains displayed reduced lifespan or increased age-related decline. GWAS implicated members of the WNT signaling pathway in the Lisinopril effect on climbing speed, and reducing the expression of some of these genes in skeletal muscle reduced Lisinopril’s beneficial impact on climbing. However, the effect of the WNT signaling pathway on Lisinopril-induced lifespan extension or other aging phenotypes was not reported.

### RAS inhibitors can increase lifespan in rodents

Rodents have long been the organism of choice for studying the RAS, given their historical use in drug discovery. Their use in the study of aging is more complicated because of their relatively long lifespan (∼3 years) compared to worms and flies, making studies of the effects of drugs on lifespan more labor-intensive and expensive.

The RAS has been a popular target of study for aging in mice and rats due to its well-studied effects on blood pressure. Several rodent models displaying spontaneous hypertension are in common use; however, in comparison to the large body of evidence examining its effects on blood pressure regulation, relatively few studies have examined the effects of the inhibition of the RAS on aging, normotensive rodents (Jama et al., 2022). In aging hypertensive rodent models, the inhibition of the RAS may increase the survival rate due to alleviation of hypertension-associated morbidity; these effects cannot be distinguished from any beneficial effects on age-related degeneration itself. Therefore, the studies discussed below are only those that use normotensive rodents, which measure the effect of RAS inhibition in aged mice and that use an untreated age-matched cohort as a control.

Genetic knockout of components of the RAS has been shown to influence aging in rodents. Benigni et al. (2009) showed that the knockout of the angiotensin II type I receptor (AGT1R) increased lifespan by 25% in mice, and a similar effect was reported by Yabumoto et al. (2015) (Figure 4C; Table 2). In addition to increased lifespan, *Agt1r* ^−/−^ mice displayed increased late-life cardiac health and mitochondrial function, as well as reduced frailty and healthier skin aging (Benigni et al., 2009; Yabumoto et al., 2015). By contrast, the knockout of ACE2 accelerates aging in mice (Takeshita et al., 2018; Nozato et al., 2019; Takeshita et al., 2020), and the knockout of ACE resulted in detrimental effects on health (Esther et al., 1996) (Table 2); these data suggest that complete knockout of ACE is harmful, whereas complete knockout of AGT1R alone shows beneficial effects.

The effect of Enalapril on aging rodents is well studied. Santos et al. (2009) showed that Enalapril treatment extends the lifespan of rats by 45% on either standard or high-fat diet (Figures 1A, 3C; Table 1) (Santos et al., 2009). It has been noted that many age-related degenerative phenotypes displayed by the cardiovascular and renal systems of normotensive mammals are similar to phenotypes observed in younger hypertensive mammals (Benetos et al., 1993; Pinaud et al., 2007). Thus, high blood pressure may accelerate the aging of the cardiovascular system. Alternatively, high blood pressure may cause pathologies that resemble age-related degeneration but are actually distinct. Treatment with anti-hypertensive medications in the absence of hypertension may have beneficial anti-aging properties (Benetos et al., 1993; Assayag et al., 1997). Cardiovascular health declines with age (Miller and Arnold, 2022) and treatment with Enalapril (González Bosc et al., 2000; de Cavanagh et al., 2003; Carter et al., 2004; Basso et al., 2007; de Cavanagh et al., 2008; Inserra et al., 2009; Carter et al., 2011) or Losartan (González Bosc et al., 2000; de Cavanagh et al., 2003; Basso et al., 2007; de Cavanagh et al., 2008; Inserra et al., 2009; Carter et al., 2011) improve many aspects of the health of aged rodents (Figure 1A; Table 1). Improvements were observed in cardiac (Inserra et al., 1995; González Bosc et al., 2000; Basso et al., 2007) and renal health (Ferder et al., 1994; de Cavanagh et al., 2003; de Cavanagh et al., 2008; Inserra et al., 2009), reduced tumor incidence (Carter et al., 2011), reduced frailty (Keller et al., 2019; de Cavanagh et al., 2008; Carter et al., 2011), and reduced age-associated hypertension (González Bosc et al., 2000; de Cavanagh et al., 2003; Carter et al., 2004; Basso et al., 2007; de Cavanagh et al., 2008; Keller et al., 2019). Enalapril, but not Losartan, reduced body weight in old age, suggesting that RAS inhibition of different targets may result in distinct phenotypes (Ferder et al., 1994; Inserra et al., 1995; González Bosc et al., 2000; Basso et al., 2007; Carter et al., 2011). Body fat and food consumption were reduced, suggesting dietary restriction as a possible mechanism (Carter et al., 2004; Carter et al., 2011). Other studies have implicated mitochondria by demonstrating an increase in mitochondrial number and function late in life and a reduction of reactive oxygen species (Ferder et al., 1994; de Cavanagh et al., 2003; de Cavanagh et al., 2008).

### Associations between a polymorphism in the ACE gene and longevity in humans

Human centenarians (>100 years of age) and supercentenarians (>110 years of age) are of interest for studies of aging due to their extreme longevity and extraordinarily healthy lives. In addition to extremely long lifespans, human centenarians exhibit fewer infectious diseases, lower inflammation and cancer rates, and reduced age-related comorbidities such as Alzheimer’s disease, cardiovascular disease, and hypertension (Willcox et al., 2008; Andersen et al., 2012; Hirata et al., 2020; Sato et al., 2021). Many studies, including several meta-analyses, investigate variants that are associated with the phenotype of extreme human longevity (Sebastiani et al., 2013; Revelas et al., 2018). One candidate is the ACE polymorphism rs4340 (Revelas et al., 2018). This polymorphism was discovered by Rigat et al. (1990) and is characterized by the presence or absence of a 287bp *Alu* repetitive element in intron 16. The allele with the *Alu* sequence is defined as I (insertion), and the allele without the Alu sequence is defined as D (deletion) (Rigat et al., 1990; Sayed-Tabatabaei et al., 2006). The D allele is most likely an ancestral version because the I allele insertion is absent from the genomes of non-human primates (Batzer et al., 1994; Li et al., 2011). The I and D alleles are not homogeneously distributed among the population (Batzer et al., 1994; Li et al., 2011): The D allele is more frequent in Africa and the Middle East, whereas the I allele is more frequent in East Asia (Li et al., 2011). This polymorphism is likely to have a direct phenotypic consequence on the level of ACE in the plasma since the level of ACE activity is increased in humans with the genotype D/D (Rigat et al., 1990). Cultured primary human endothelial cells with an I/I genotype exhibited lower Ang II levels and a higher cell viability compared to cells with the D/D genotype. D/D cells phenocopied I/I cells after the addition of Captopril, indicating that the phenotype is caused by alterations in ACE activity (Hamdi and Castellon, 2004).

Several studies investigated the association of the ACE I/D polymorphism with pathologies. The D allele is positively associated with hypertension, arteriosclerosis, cardiovascular disease, and diabetic microvascular disorders and is negatively associated with Alzheimer’s disease (Sayed-Tabatabaei et al., 2006). By contrast, the I allele is associated with a higher expression of ACE2, which has been shown to negatively affect health (Delanghe et al., 2020). The positive association of the D allele with extreme longevity was investigated in over 32 studies (Human Ageing Genomic Resources, 2022). Two meta-analysis included (1) 12 studies with a total of 1803 centenarians and 10,485 non-centenarian controls (Garatachea et al., 2013), and (2) eight studies with 2043 individuals over 85 years of age and 8,820 younger controls (Revelas et al., 2018). These studies identified a positive association between increased longevity and the presence of the D allele. The authors speculate that the D allele has a negative impact early in life and may grant a survival advantage in later life by effecting tissue repair and activating the immune system, thus representing a pleiotropic effect in favor of longevity (Revelas et al., 2018). It is important to note that studies with centenarians are cross-sectional rather than longitudinal, and the control group is younger individuals from a different birth cohort. In addition, associations might be caused by nearby polymorphisms that are linked to the D or I allele.

Currently available results suggest the possibility that ACE may have undefined functions in addition to its effect on blood pressure that may be responsible for the longevity effects. Humans treated with ACE inhibitors or ARBs display improvements in some measures of health and a reduction in all-cause mortality; however, no aging-focused study has been performed on normotensive humans treated with these drugs (Zoja et al., 2010; Kwang Chae et al., 2011; Abdel-Zaher et al., 2014; Faglia et al., 2014). Interestingly, reduced mortality was reported in diabetic patients treated with ACE inhibitors but not in patients treated with the cholesterol-lowering medication Statin, suggesting that the effect of ACE inhibitors on human health is at least partially independent of its effect on cardiovascular health (Faglia et al., 2014). Future studies should address the mechanistic basis of ACE-associated longevity with a focus on determining to what degree this longevity is caused by a reduction in blood pressure.

## Conclusions and perspectives

The large body of research performed on the RAS over the last several decades makes it clear that the RAS can significantly influence aging. However, several important questions at the intersection of the RAS and aging remain unanswered.

What is the function of the renin–angiotensin system in general, and the angiotensin-converting enzyme in particular, in non-vertebrate animals that lack closed circulatory systems? Treating hypertensive humans with medicines that reduce blood pressure makes them live longer by alleviating the pathologies caused by high blood pressure, including strokes and heart disease. Thus, one model for how these same drugs extend lifespan is by reducing blood pressure. However, RAS inhibition extends lifespan in normotensive rodents. While this might indicate that lowering blood pressure below the normal level extends lifespan, it also hints that there is another mechanism. Furthermore, how does the inhibition of ACE affect aging in animals that lack a closed circulatory system altogether? Clearly, in these animals, the effect cannot be on blood pressure, so there must be an alternative mechanism. Although *Drosophila* has tissues that are analogous to the vertebrate cardiac and pulmonary systems, *C. elegans* lacks these systems altogether, yet ACE inhibitors extend lifespan in both of these organisms. We speculate that ACE has some ancestral function that mediates its effect on aging.

It is well established that ACE is an essential enzyme in many organisms; the knockout of ACE or its homologs in other species has severe negative effects on health in mammals and is lethal in *Drosophila* and *C. elegans* (Tatei et al., 1995; Esther et al., 1996; Brooks et al., 2003; Kumar et al., 2016; Nichols et al., 2016). ACE likely evolved in a hypothesized most recent common ancestor of the Bilaterian clade; since that time functional ACE homologs have been retained in such diverse phyla as insects (Lamango and Isaac, 1994; Cornell et al., 1995; Wijffels et al., 1996; Wijffels et al., 1997; Loeb et al., 1998; Isaac et al., 1999; Vandingenen et al., 2001; Vandingenen et al., 2002; Ekbote et al., 2003a; Ekbote et al., 2003b; Nathalie et al., 2003; Burnham et al., 2005; Lemeire et al., 2008; Wang et al., 2015; Abu Hasan et al., 2017; Nagaoka et al., 2017; Wang et al., 2019), crustaceans (Smiley and Doig, 1994; Kamech et al., 2007; Sook Chung and Webster, 2008), mollusks (Laurent et al., 1997; Riviere et al., 2011), annelids (Rivière et al., 2004), nematodes (Brooks et al., 2003; Kumar et al., 2016; Metheetrairut et al., 2017; Kucuktepe, 2021), and vertebrates (reviewed in (Lv et al., 2018)). Metalloprotease activity, predicted by the highly conserved histidine-rich HEXXH motif, is retained in all known organisms with an active ACE other than nematode ACN-1, indicating a high degree of evolutionary selective pressure to retain this motif. ACE inhibitors bind to and competitively inhibit this active site, and ACE inhibitors have been shown to function in non-vertebrate animals (Lamango and Isaac, 1994; Smiley and Doig, 1994; Wijffels et al., 1996; Wijffels et al., 1997; Isaac et al., 1999; Vandingenen et al., 2001; Vandingenen et al., 2002; Ekbote et al., 2003a; Ekbote et al., 2003b; Nathalie et al., 2003; Rivière et al., 2004; Vercruysse et al., 2004; Kamech et al., 2007; Lemeire et al., 2008; Sook Chung and Webster, 2008; Nagaoka et al., 2017). ACE has been shown to be involved in fertility in mice, *Drosophila,* and other arthropods (Wijffels et al., 1996; Wijffels et al., 1997; Loeb et al., 1998; Ramaraj et al., 1998; Isaac et al., 1999; Vandingenen et al., 2002; Ekbote et al., 2003a; Ekbote et al., 2003b; Hurst et al., 2003; Nathalie et al., 2003; Vercruysse et al., 2004; Kamech et al., 2007; Sook Chung and Webster, 2008; Riviere et al., 2011; Nagaoka et al., 2017), being commonly expressed in the testis or ovaries, and effecting sperm motility or progeny production in many species. This is especially interesting considering the existence of a testis-specific isoform of ACE in mammals, called tACE; this isoform has been shown to regulate male fertility and sperm function (Hagaman et al., 1998). ACE has also been implicated in ecdysis (Ekbote et al., 2003a; Ekbote et al., 2003b; Vercruysse et al., 2004; Lemeire et al., 2008; Metheetrairut et al., 2017), being most strongly expressed during larval molts in several species. Thus, ACE plays many roles in many different organisms, but it is likely that its role as a blood pressure regulator was a later development, as this activity is not observed outside of vertebrates. It is likely that ACE evolved from earlier peptidyl dipeptidases and diverged to serve many functions; one later development would be the maturation of blood pressure signaling peptides in a vertebrate ancestor, whose function was retained in modern vertebrates. It is likely, then, that any potential secondary effects on aging were retained in mammals as well, explaining the seemingly dual effects of ACE inhibitors on aging and blood pressure.

The *C. elegans* homolog of ACE does not conserve some amino acids necessary for the catalytic function, leading to the model that it is not an active enzyme (Brooks et al., 2003). However, it has an important function because the genetic knockout of *acn-1* is lethal and it has been implicated as an essential regulator of molting and development, indicating an essential role for *acn-1* despite the predicted lack of metalloprotease activity (Brooks et al., 2003; Kumar et al., 2016; Metheetrairut et al., 2017). Kumar et al. (2016) demonstrated that Captopril treatment or knockdown of *acn-1* by RNAi still resulted in lifespan extension if administered after the final larval molt, once all known functions of *acn-1* have presumably been completed. What is the function of *acn-1* in adulthood, and why does inhibiting this function affect aging? It is possible that ACN-1 merely sequesters or is involved in the maturation of its substrate rather than acting through an enzymatic activity, or that it acts in concert with a second enzyme. Further research must be done to determine what, if any, substrate is associated with ACN-1.

The discovery of drugs that influence aging has traditionally been a strength of non-vertebrate model organisms; *C. elegans* in particular has been a fruitful ecosystem for repurposing well-studied human drugs into potential anti-aging treatments. Two of the most well-studied anti-aging compounds are the FDA-approved medications Metformin and Rapamycin. Metformin was first developed to treat type 2 diabetes, and Rapamycin was approved as an immunosuppressant for organ transplants. Subsequent research in *C. elegans* (Onken and Driscoll, 2010; Robida-Stubbs et al., 2012; Cabreiro et al., 2013) and *Drosophila* (Bjedov et al., 2010) demonstrated that these drugs control aging. Metformin has been shown to control aging in nematodes and mice (reviewed in (Glossmann and Lutz, 2019; Hu et al., 2021)). Additionally, the Targeting Research with MEtformin (TAME) project is currently testing the effect of Metformin on mortality in elderly humans (Justice et al., 2018). The National Institute of Aging’s Interventions Testing Program (ITP) has identified Rapamycin as a potent controller of aging, extending lifespan in both male and female mice by more than 10% (Harrison et al., 2009; Miller et al., 2011). Rapamycin has also been tested as a potential anti-aging therapy in non-human primates (Ross et al., 2015) and is currently being tested in canines (Kaeberlein et al., 2016). Captopril is currently undergoing testing by the ITP as well and is on track to become the third FDA-approved drug with potential as an anti-aging therapy. Captopril is an ideal candidate for future human studies due to its long history as a safe, effective blood pressure medication, but it has not yet been tested as an anti-aging therapy in normotensive humans. Future research in model organisms will lay the foundation for testing Captopril and other RAS inhibitors for use as an anti-aging therapy in humans.

## Abbreviations

ACE, Angiotensin-converting enzyme; ACE2, Angiotensin-converting enzyme 2; Acer, Angiotensin-converting enzyme-related; AGT1R, Angiotensin II type-1 receptor; AGT2R, Angiotensin II type-2 receptor; Ance, ANgiotensin-converting enzyme; AGT, Angiotensinogen; Ang I, Angiotensin I; Ang II, Angiotensin II; ARB, Angiotensin II type-1 receptor blocker; DR, Dietary restriction; GWAS, Genome-wide association study; GFP, Green fluorescent protein; IGF, Insulin-like growth factor; ITP, Interventions Testing Program; RAS, Renin–angiotensin system; RNAi, RNA interference; TOR, Target of Rapamycin.
